# Supplementary material for: SARS-CoV-2 hijacks folate and one-carbon metabolism for viral replication
Source: Nat Commun. 2021 Mar 15;12:1676. doi: 10.1038/s41467-021-21903-z (PMC7960988; doi:10.1038/s41467-021-21903-z)
Supplement: Supplementary file 1 — Supplementary Information [file 41467_2021_21903_MOESM1_ESM.pdf]

Supplementary Fig. 1

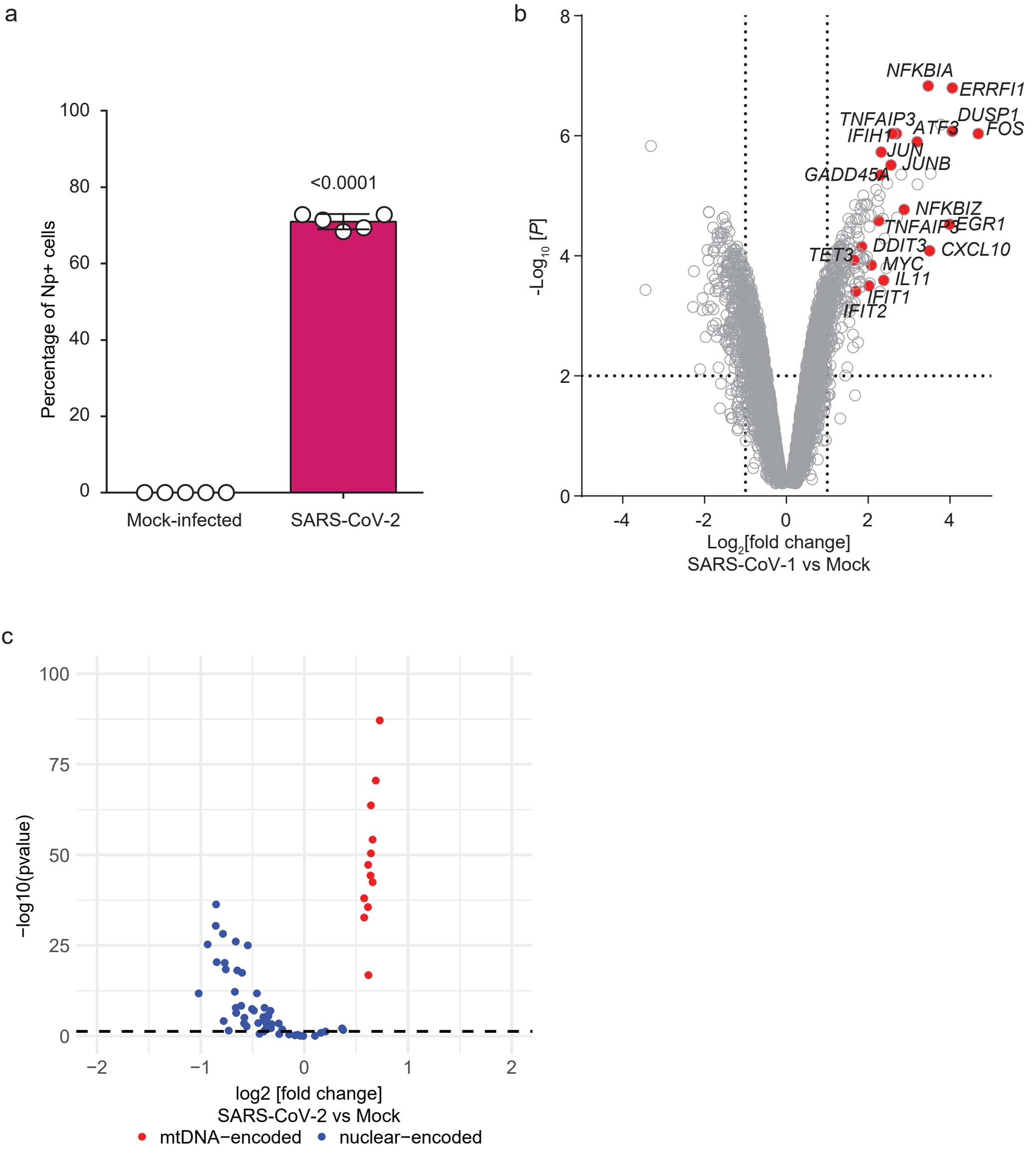

Supplementary Figure 1. Metabolite analysis of SARS-CoV-2 infected Vero E6 TMRPSS2+ and transcriptome analysis of SARS-CoV-1 infected Vero E6 cells at 7 hpi.

a, Mean ± SD percentages of Np+ cells as in Fig. 1a-b, from 1475 cells, obtained from 5 random fields for each condition. P-values were calculated by two tailed unpaired Student's t-test.

b, Volcano plot visualization of -Log10 (p-value) statistical significance (y-axis) and Log2 fold-change of mRNA abundance in SARS-CoV-1 versus mock infected cells (x-axis) from triplicate microarray datasets[13]. Values for selected SARS-CoV-1 induced genes are highlighted in red and labeled.

c, Volcano plot visualization of -Log10 (p-value) statistical significance (y-axis) and Log2 fold-change of mRNA abundance in SARS-CoV-2 versus mock infected cells (x-axis), as in Fig. 1c. mtDNA-encoded OXPHOS subunits were labeled as red circles. Nuclear-encoded OXPHOS subunits were labeled as blue circles.

Supplementary Fig. 2

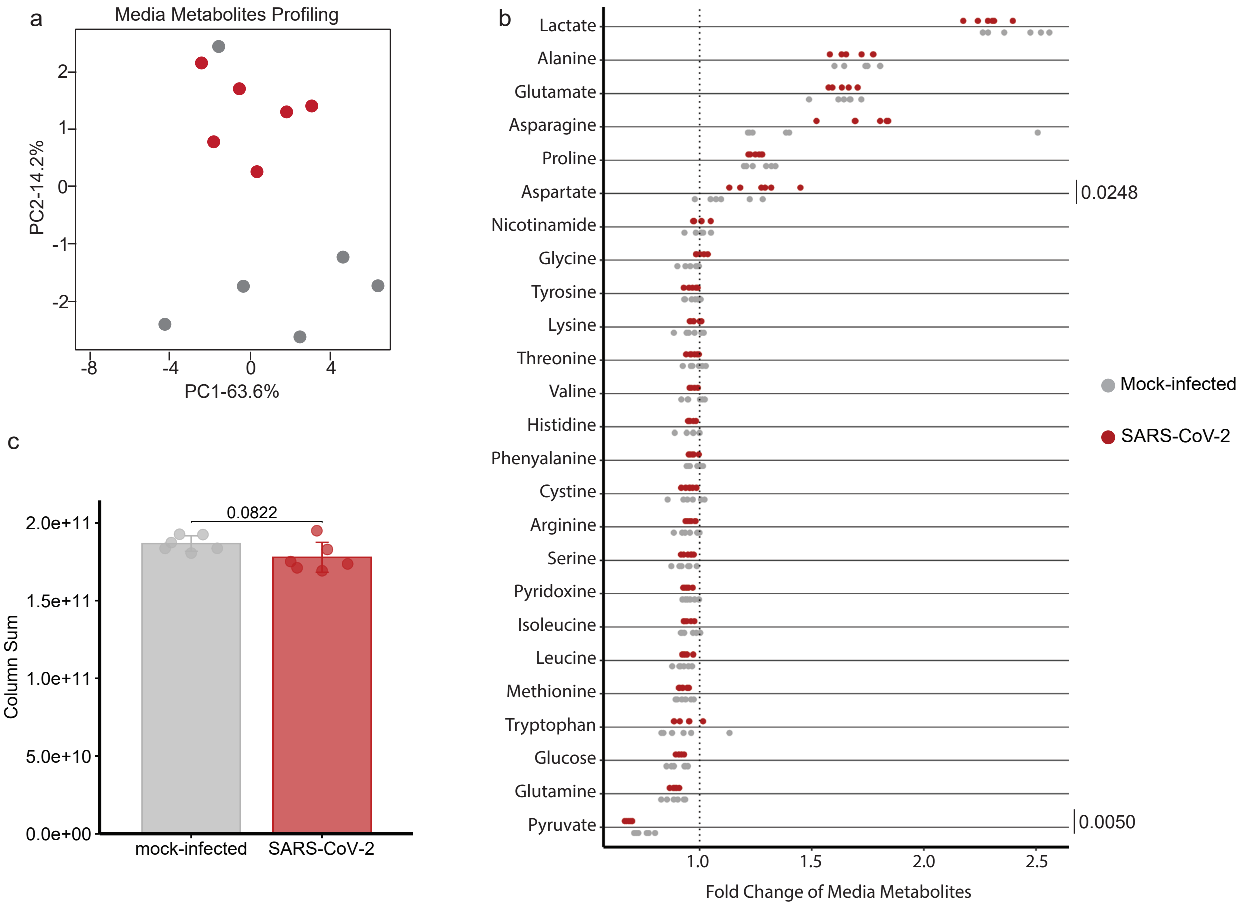

Supplementary Figure 2. Quantitative media metabolite profiling and sum of all metabolite signals of mock or SARS-CoV-2 infected Vero E6 TMPRSS2+ cells at 8 hpi.

a, PCA of 25 spent media metabolites, as determined by LC-MS of n=6 biologically independent replicates.

b, Quantitative analyses of fold-change of select media metabolites in SARS-CoV-2 infected (red) versus mock-infected Vero E6 TMPRSS2+ cells (gray) from n=6 biologically independent replicates. P-values were generated with two-tailed unpaired Student's t-test assuming unequal variance, comparing production or consumption of each metabolite.

c, Column sum, the sum of detected known metabolite signal, for mock and SARS-CoV-2 infected Vero E6 TMPRSS2+, as in Fig. 1a-b. n=6 biologically independent samples were examined over 1 independent experiment.

Supplementary Fig. 3

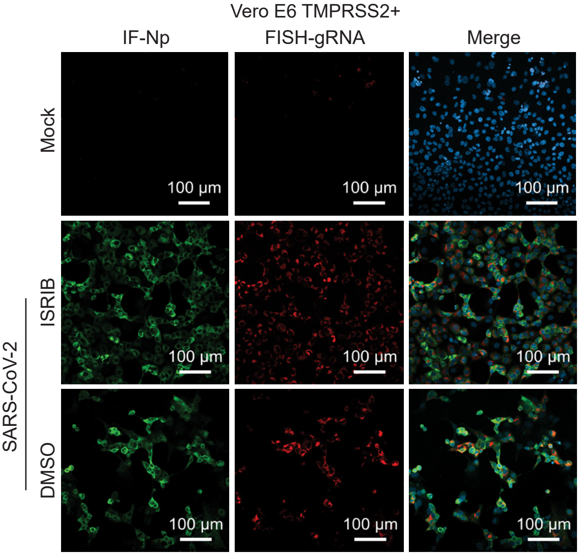

Supplementary Figure 3. Analysis of ISR inhibitor effects on SARS-CoV-2 infection.

IF of Np, FISH for +strand gRNA and merge with Hoechst stained nuclei in mock or SARS-CoV-2 infected Vero E6 TMPRSS2+ cells treated with DMSO or 10μM of ISRIB, a small molecule integrated stress response inhibitor for 48 hours. Images are representative of n=3 biologically independent replicates. P-values were calculated by two tailed unpaired Student t test.

Supplementary Fig. 4

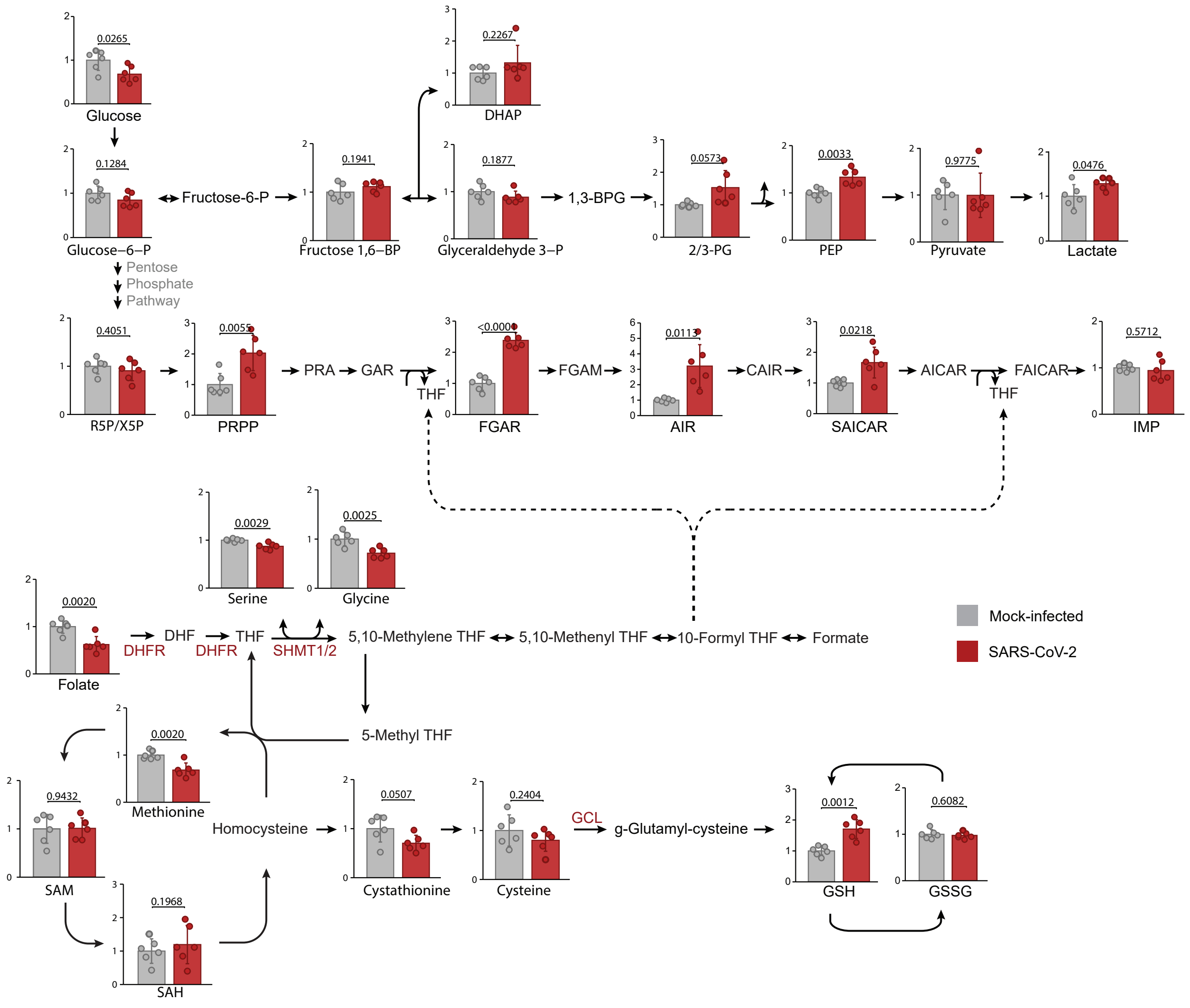

Supplementary Figure 4. Intracellular glucose, lactate, de novo purine and one-carbon metabolite levels normalized to mock-infected Vero E6 TMPRSS2+ cell levels.

Druggable targets are labeled in red. All barplots show mean ± SD from n=6 biologically independent replicates. **P*<0.05, ***P*<0.01, or ****P*<0.001 from Student's two-tailed t-test.

Supplementary Fig. 5

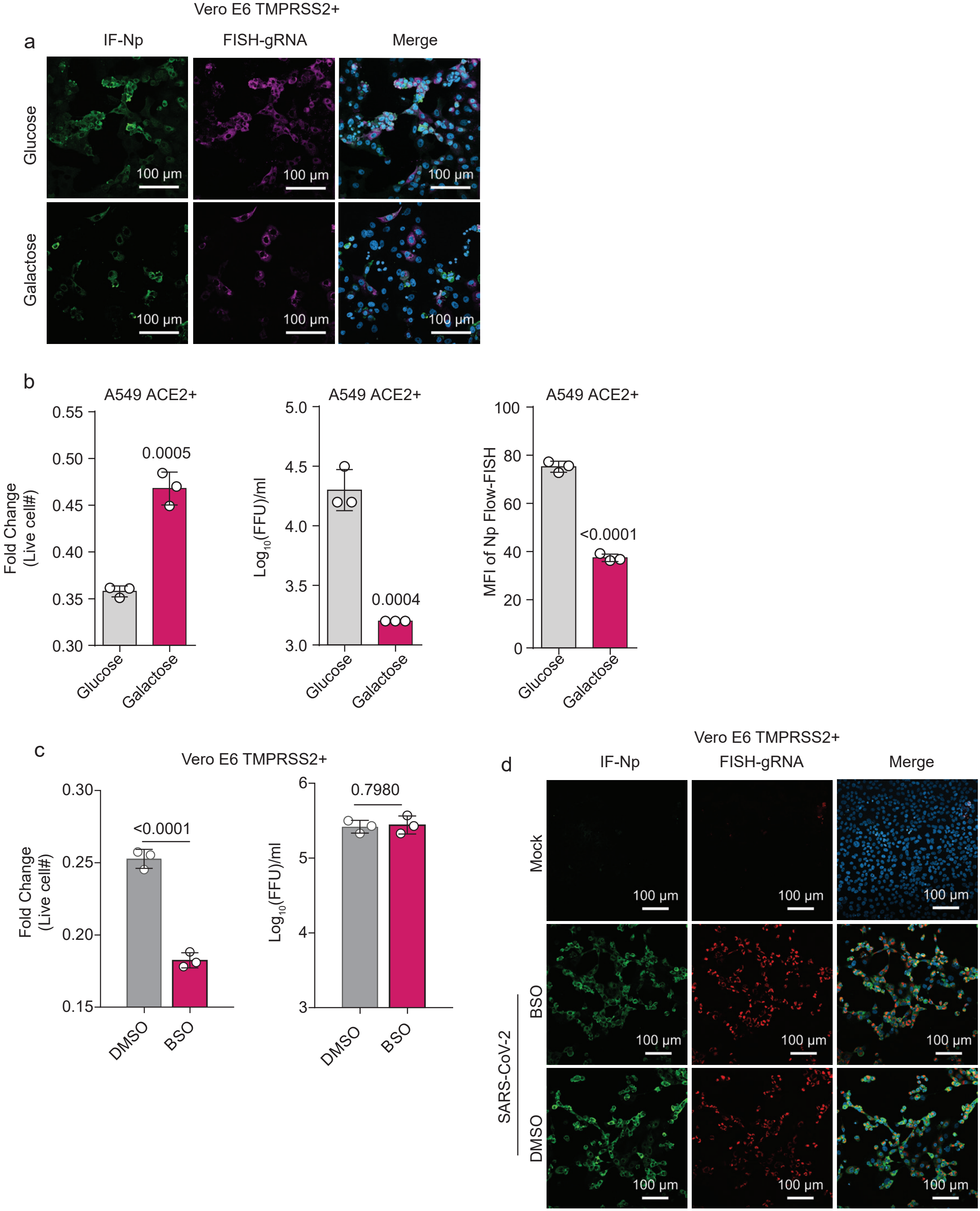

Supplementary Figure 5. Analysis of the effects of glucose deprivation, complex I inhibition, or glutathione inhibition on SARS-CoV-2 infection.

a, IF of Np, FISH for +strand gRNA and merge with Hoechst stained nuclei in mock or SARS-CoV-2 infected Vero E6 TMPRSS2+ cells cultured in media with 25 mM glucose or galactose for 48 hours.

b, Fold change mean ± SD live cell number, TCID50 virus titer, and Flow-FISH analysis of Np sub-genomic RNA at 48 hpi of A549 ACE2+ cells cultured in media with 25mM glucose versus galactose as the sugar source, from n=3 biologically independent replicates.

c, Fold change mean ± SD live cell (left) and TCID50 virus titer (right) from n=3 biologically independent replicates of mock or SARS-CoV-2 infected Vero E6 TMPRSS2+ cells treated for 48 hours with DMSO or 10μM of BSO, an inhibitor of glutathione synthesis.

d, IF of Np, FISH for +strand gRNA and merge with Hoechst stained nuclei in mock or SARS-CoV-2 infected Vero E6 TMPRSS2+ cells treated with DMSO or 10μM BSO for 48 hours.

In all panels, cells were infected at MOI=0.1 for 48 hours. Microscopy images are representative of at least n=3 biologically independent values. P-values in this figure were calculated by one-way ANOVA with multiple comparisons using Sidak method.

Source data are provided as a Source Data file

Supplementary Fig. 6

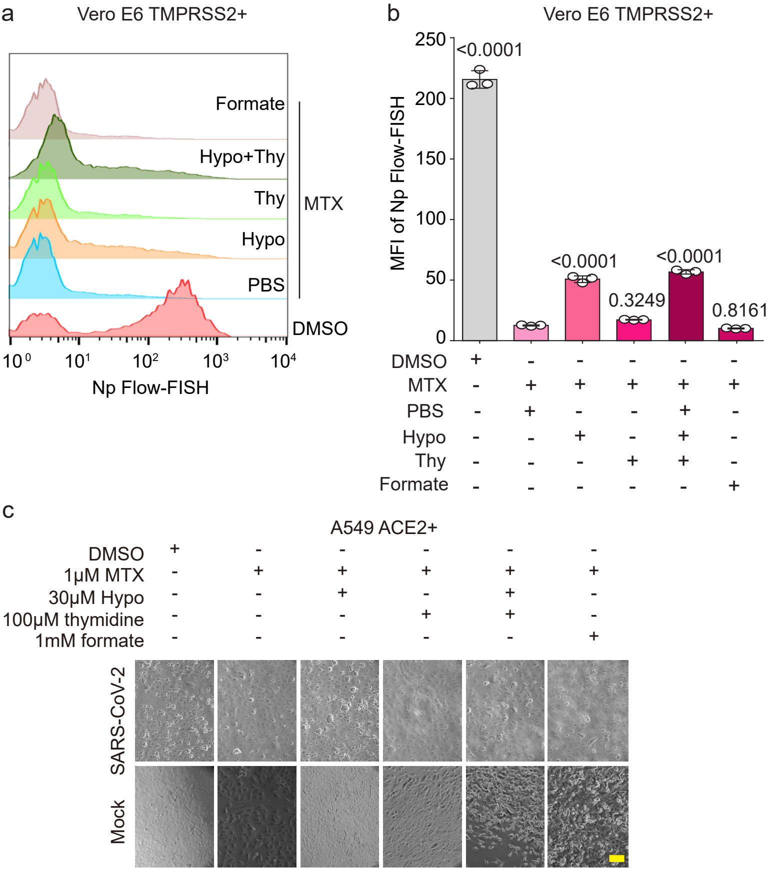

Supplementary Figure 6. Flow-FISH analysis of methotrexate and metabolite rescue effects on Np subgenomic RNA expression and viral cytopathic effect.

a, Representative Np subgenomic RNA Flow-FISH plots of Vero E6 TMPRSS2+ cells infected by SARS-CoV-2 at MOI=0.1 and cultured for 48 hours with DMSO, 1μM of methotrexate (MTX), 30 μM hypoxanthine (hypo), 100 μM thymidine, 1mM formate, as indicated. The leftmost peak of each row indicates uninfected cells that do now express Np subgenomic RNA.

b, Mean ± SD of Np subgenomic RNA MFI values from n=3 biologically independent replicates, as in (a). P-values were calculated by one-way ANOVA with multiple comparisons using Sidak method.

c, Phase microscopic images of SARS-CoV-2 versus mock infected A549 ACE2+ cells cultured for 48 hours with DMSO, 1μM of methotrexate (MTX), 30 μM hypoxanthine (hypo), 100 μM thymidine, or 1mM formate, as indicated. Yellow scale bar indicates 100 μm. The experiment was reproduced in 3 independent experiments.

Source data are provided as a Source Data file

Supplementary Fig. 7

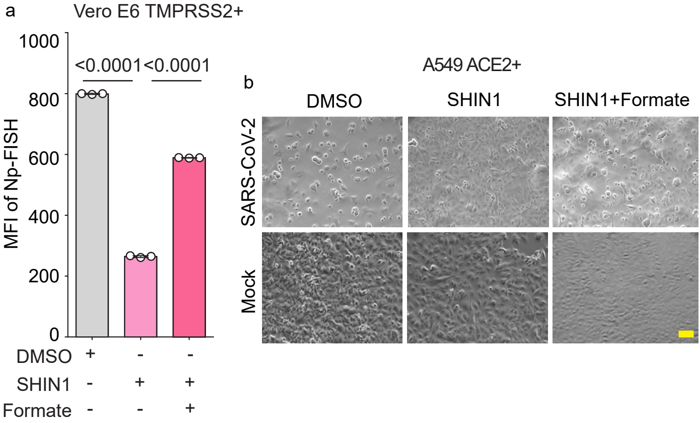

Supplementary Figure 7. SHIN1 and formate rescue effects on Np subgenomic RNA expression and viral cytopathic effect.

a, Mean ± SD of Np subgenomic RNA Flow-FISH MFI values from n=3 biologically independent replicates of Vero E6 TMPRSS2+ cells infected by SARS-CoV-2 at MOI=0.1 and cultured for 48 hours with DMSO, 10μM of SHIN1 or 1mM formate as indicated. P-values were calculated by one-way ANOVA with multiple comparisons using Sidak method.

b, Phase microscopic images of SARS-CoV-2 versus mock infected A549 ACE2+ cells cultured with DMSO, 10 μM of the dual SHMT1/2 inhibitor SHIN1 or 10 μM SHIN1 + 1 μM formate, as indicated. Yellow scale bar indicates 100 μm. The experiment was reproduced in 3 independent experiments.

Source data are provided as a Source Data file

Supplementary Fig. 8

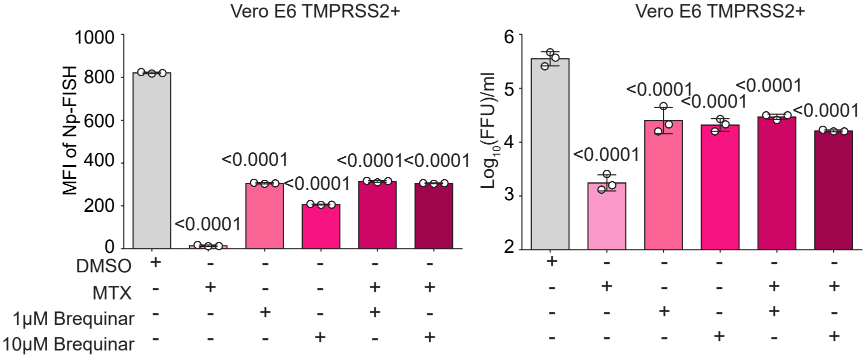

Supplementary Figure 8. *De novo* pyrimidine pathway role in SARS-CoV-2 infection.

Mean ± SD fold change live cell # (left), Np subgenomic RNA Flow-FISH MFI (middle) and TCID50/ml viral load (right) values from Vero-E6 TMPRSS2+ cells treated with DMSO, 1μM MTX, 1 or 10 μM of the DHODH inhibitor brequinar, as indicated from two hours pre-infection and infected with SARS-CoV-2 at MOI=0.1 for 48 hours. Data are from n=3 biologically independent replicates. P values were calculated by one-way ANOVA, Sidak post-test.

Supplementary Fig. 9

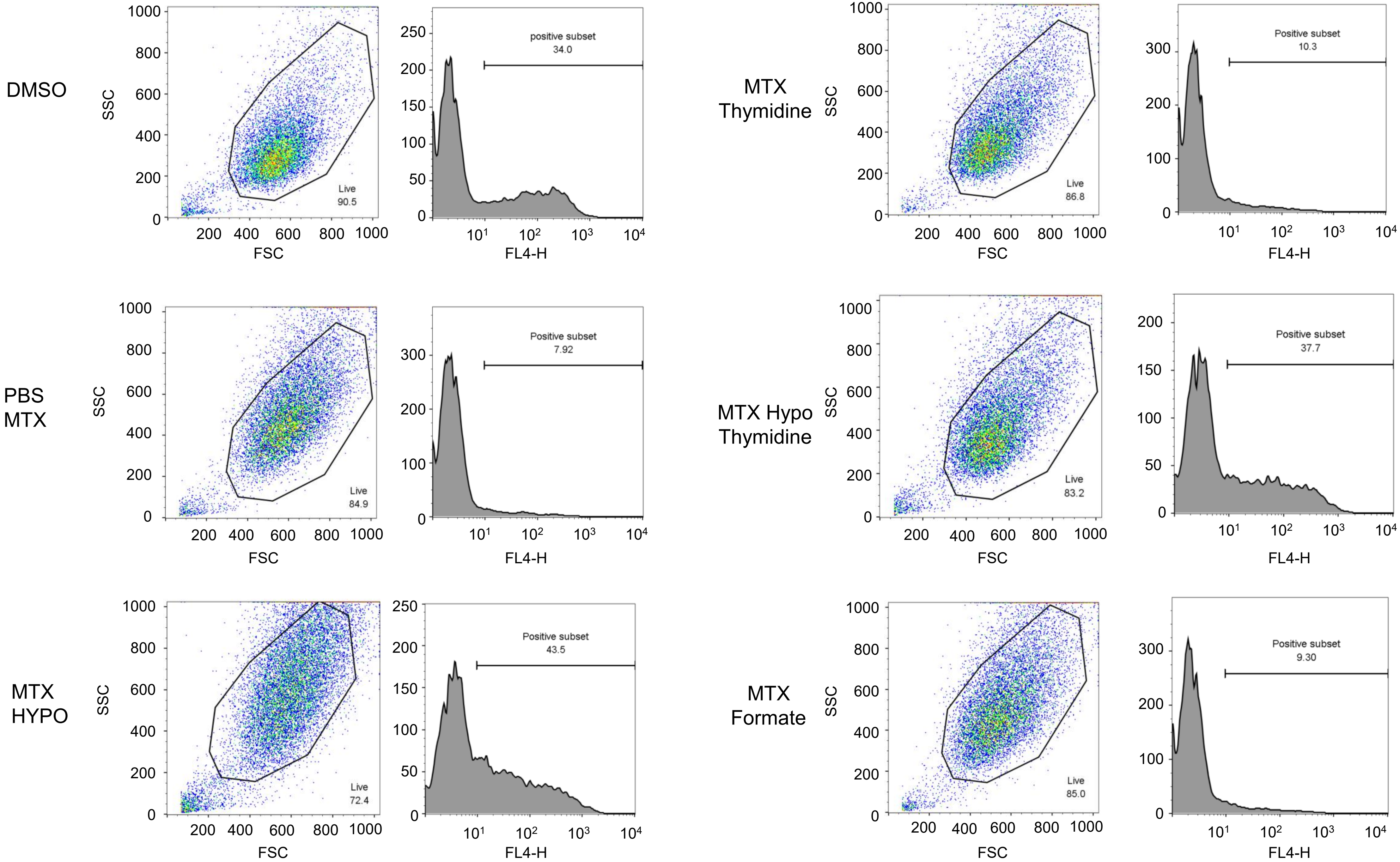

Fig.2I A549 ACE2+ cells

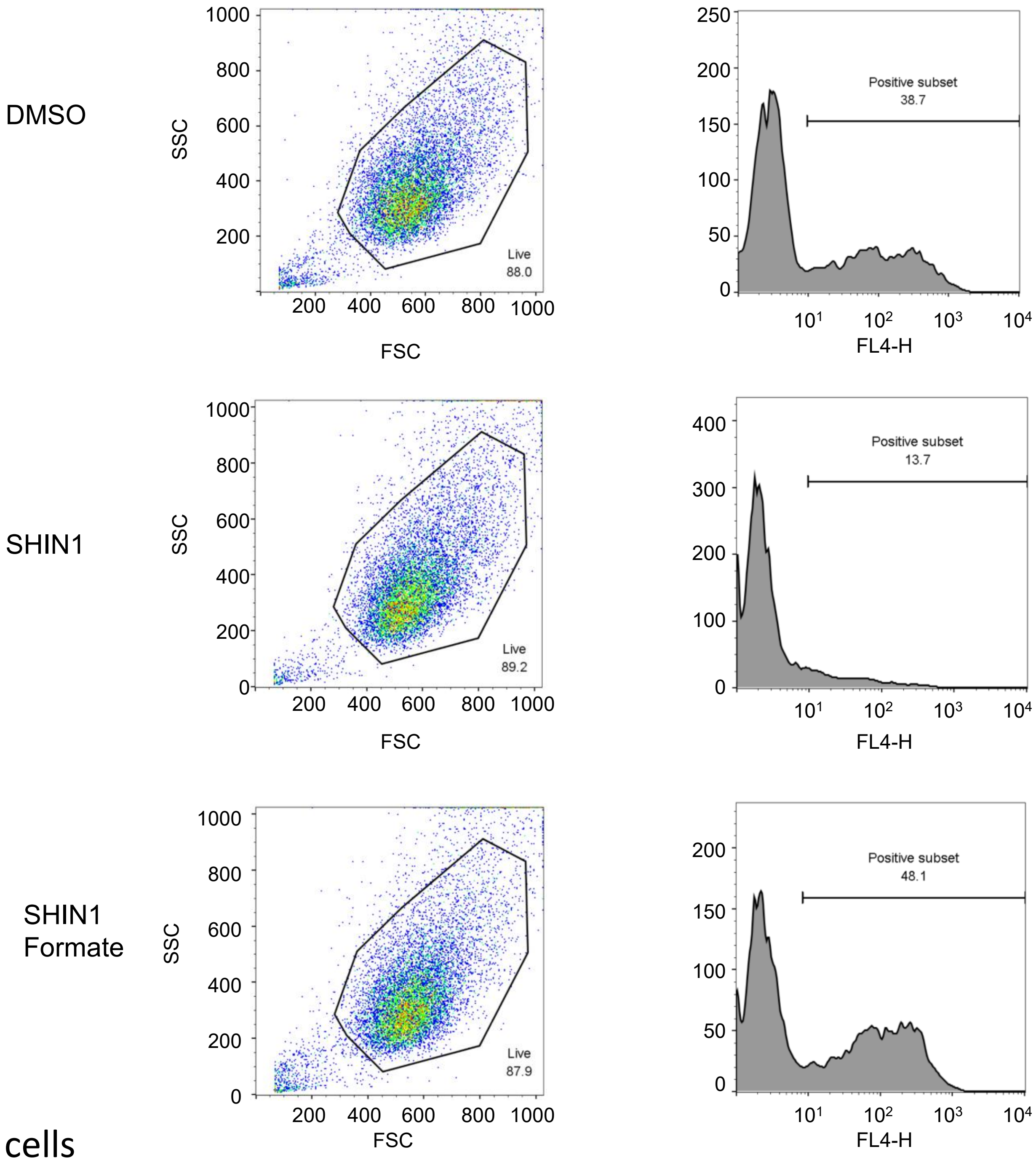

Fig.3g A549 ACE2+ cells

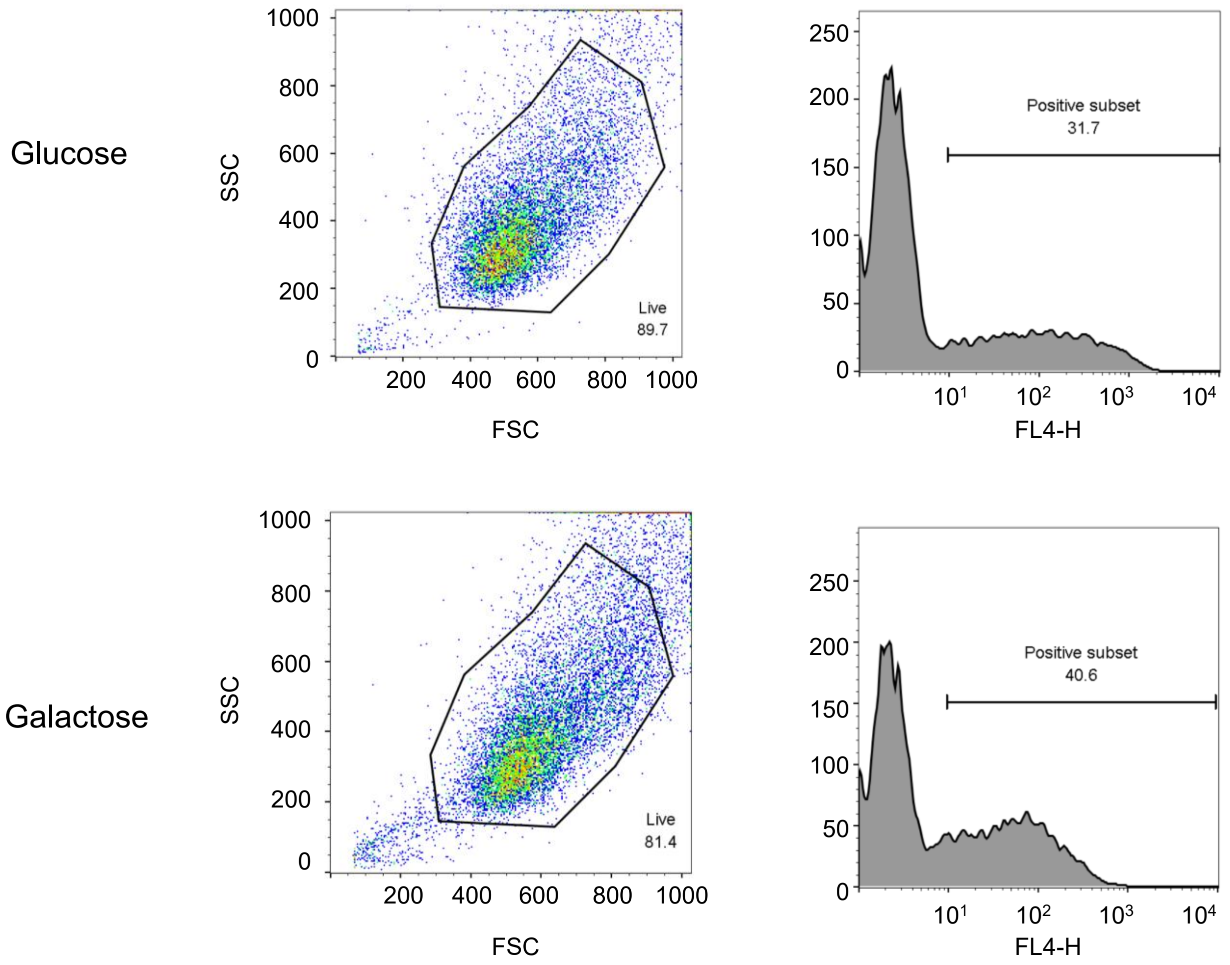

Fig.S5b A549 ACE2+

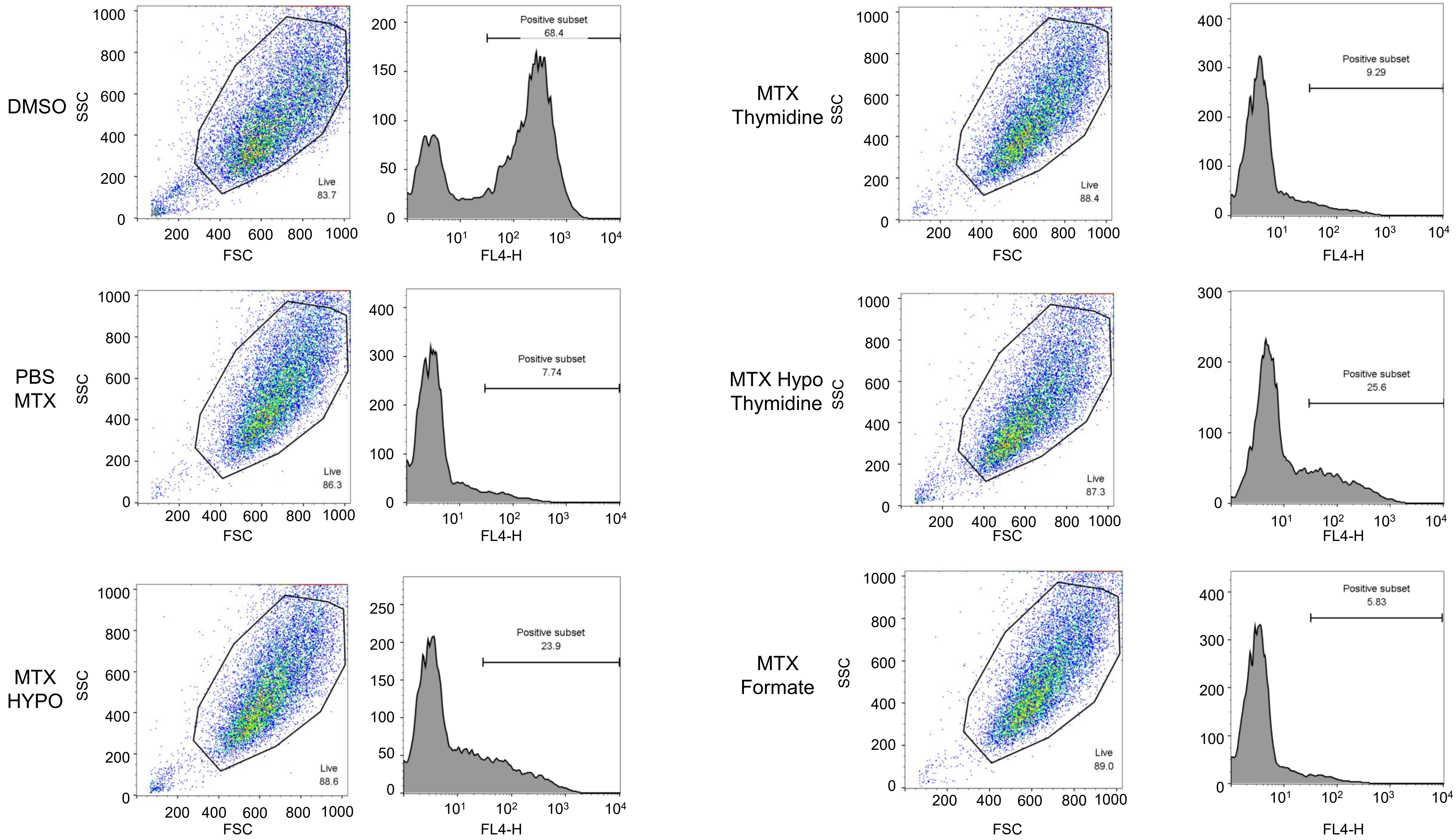

Supplementary Fig6b Vero E6 TMPRSS2+

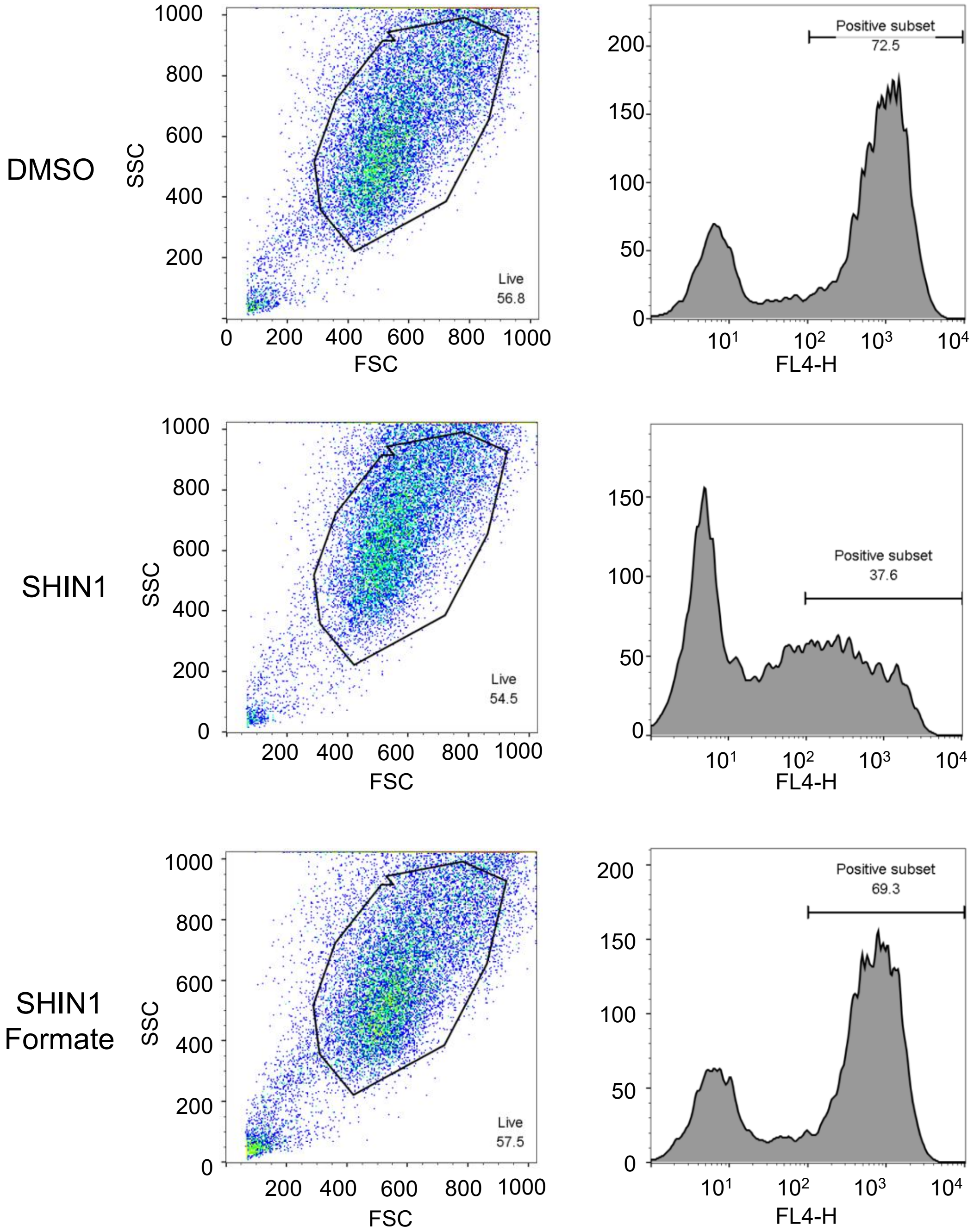

Supplementary Fig. 7a Vero E6 TMPRSS2+

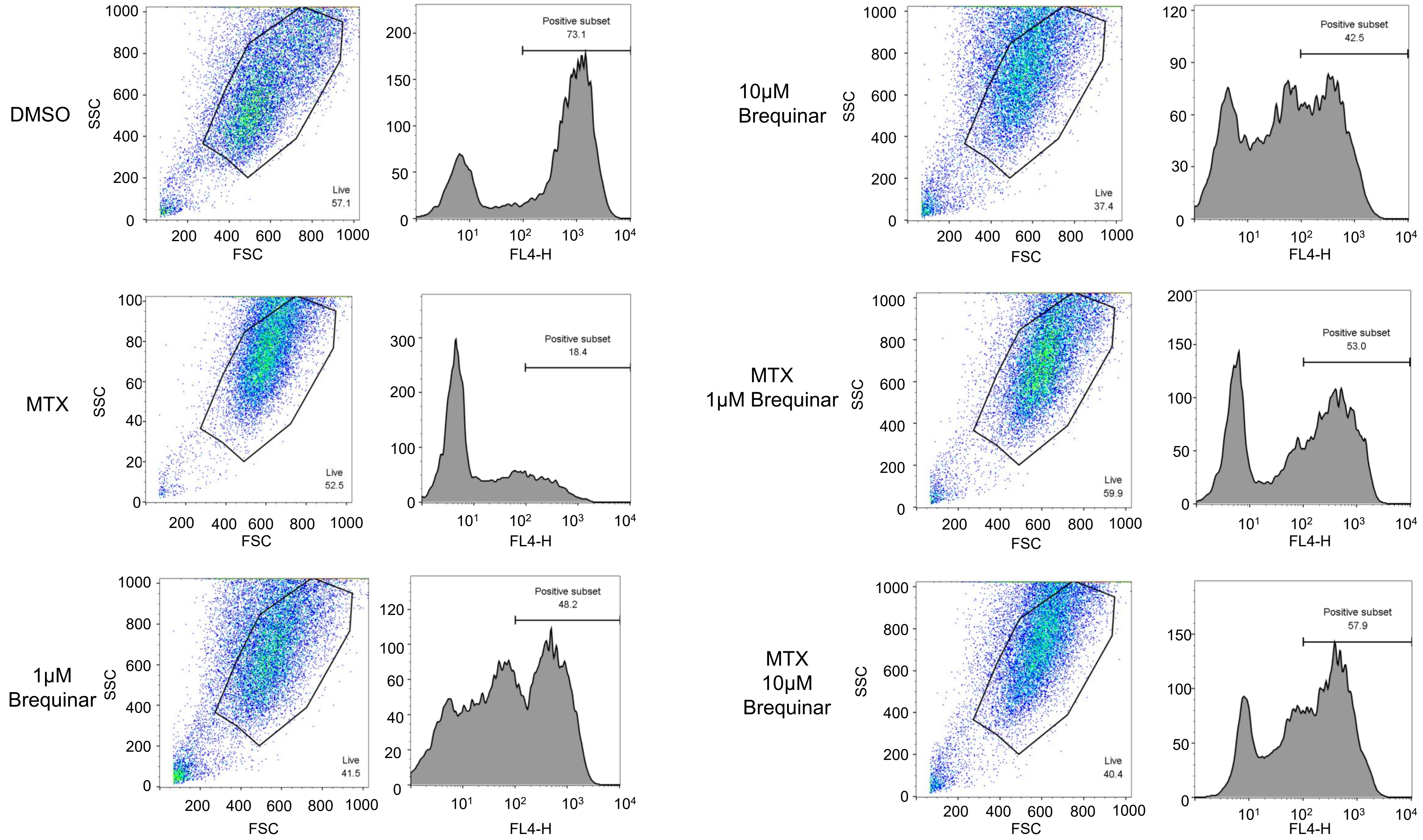

Supplementary Fig. 8 Vero E6 TMPRSS2+ cells

Supplementary Figure 9. FACS gating strategy

Live cells were gated by forward scatter (FSC) and side scatter (SSC) gates as shown throughout. For Fig. 2I, 3g and Supplementary Fig.5B, A549 ACE2+ cells were analyzed by FACS, using the gates shown. For A549 ACE2+ cell Np RNA Flow-FISH analysis, uninfected cell peaks were positioned between 0 to 10^1 as shown. A549 ACE2+ SARS-CoV-2 infected cells were gated from >10^1 MFI as shown, using channel FL4. In Supplementary Figs. 6a-b, 7a and 8, Vero E6 TMPRSS2 cells were likewise analyzed by Np RNA Flow-FISH. Live cells were gated by forward scatter (FSC) and side scatter (SSC) gates as shown throughout. Uninfected cell peaks were positioned between 0 to 10^1 as shown, and infected cell peaks were gated from >10^2 MFI as shown, using channel FL4.
