# Supplementary material for: SARS-CoV-2 hijacks folate and one-carbon metabolism for viral replication
Source: Nat Commun. 2021 Mar 15;12:1676. doi: 10.1038/s41467-021-21903-z (PMC7960988; doi:10.1038/s41467-021-21903-z)
Supplement: Supplementary file 3 — Description of Additional Supplementary Files [file 41467_2021_21903_MOESM3_ESM.pdf]

Description of Additional Supplementary Files

File Name: Supplementary Data 1
Description: RNAseq analysis of SARS-CoV-2 infected versus mock infected Vero-TMPRSS2+ cell at 8 hpi. n=3 biologically independent samples were examined. The P value and log fold change in the table were generated with DESeq2 under default settings with Wald test and normal shrinkage, respectively.

File Name: Supplementary Data 2
Description: Quantitative spent media analyses of SARS-CoV-2 infected versus mock infected Vero E6 TMPRSS2+ cell at 8 hpi. n=6 biologically independent samples were examined. P-values were generated with two-tailed P value from Student's t-test.

File Name: Supplementary Data 3
Description: Intracellular metabolite profiling of SARS-CoV-2 infected versus mock infected Vero E6 TMPRSS2+ cell at 8 hpi. n=6 biologically independent samples were examined. P-values were generated with two-tailed P value from Student's t-test.

File Name: Supplementary Data 4
Description: . Oligonucleotides used in the study.
