# Supplementary material for: SARS-CoV-2 hijacks folate and one-carbon metabolism for viral replication
Source: Nat Commun. 2021 Mar 15;12:1676. doi: 10.1038/s41467-021-21903-z (PMC7960988; doi:10.1038/s41467-021-21903-z)
Supplement: Supplementary file 8 — Reporting Summary [file 41467_2021_21903_MOESM8_ESM.pdf]

nature research

Corresponding author(s): Benjamin Gewurz
Vamsi K. Mootha

Last updated by author(s): 2021-1-8

Reporting Summary

Nature Research wishes to improve the reproducibility of the work that we publish. This form provides structure for consistency and transparency in reporting. For further information on Nature Research policies, see our Editorial Policies and the Editorial Policy Checklist.

Statistics

For all statistical analyses, confirm that the following items are present in the figure legend, table legend, main text, or Methods section.

n/a	Confirmed	
☐	☒	The exact sample size (*n*) for each experimental group/condition, given as a discrete number and unit of measurement
☐	☒	A statement on whether measurements were taken from distinct samples or whether the same sample was measured repeatedly
☐	☒	The statistical test(s) used AND whether they are one- or two-sided<br>*Only common tests should be described solely by name; describe more complex techniques in the Methods section.*
☒	☐	A description of all covariates tested
☐	☒	A description of any assumptions or corrections, such as tests of normality and adjustment for multiple comparisons
☐	☒	A full description of the statistical parameters including central tendency (e.g. means) or other basic estimates (e.g. regression coefficient) AND variation (e.g. standard deviation) or associated estimates of uncertainty (e.g. confidence intervals)
☐	☒	For null hypothesis testing, the test statistic (e.g. F, t, r) with confidence intervals, effect sizes, degrees of freedom and P value noted<br>*Give P values as exact values whenever suitable.*
☒	☐	For Bayesian analysis, information on the choice of priors and Markov chain Monte Carlo settings
☒	☐	For hierarchical and complex designs, identification of the appropriate level for tests and full reporting of outcomes
☒	☐	Estimates of effect sizes (e.g. Cohen's *d*, Pearson's *r*), indicating how they were calculated

Our web collection on statistics for biologists contains articles on many of the points above.

Software and code

Policy information about availability of computer code

Data collection	FACS data were acquired by BD FACSCalibur using Cell Analyzer Software. Western blot data were acquired by Licor Odyssey Fc Imaging System using software Image Studio ver. 4.0. Confocal microscopy data were acquired by Zeiss LSM 800 instrument using the Zeiss ZEN Blue software. Metabolite profiling was performed using Dionex Ultimate 3000 UHPLC system coupled to Q-Exactive plus orbitrap mass spectrometer (ThermoFisher Scientific, Waltham, MA) with an Ion Max source and HESI II probe operating in switch polarity mode. The model was drawn on the Biorender website. Barcharts and volcano plots were generated with ggplot2 in R.
Data analysis	The FACS data were further analyzed with FlowJo (version V10). or RNAseq analysis, adaptor-trimmed Illumina reads for each individual library were mapped back to the human GRCh37.83 transcriptome assembly using STAR2.5.2b. DESeq2 was used to evaluate differential expression (DE). Metabolomics analysis was performed by Xcalibur 4.1 (ThermoFisher Scientific) and performed in full scan mode with a range of 70-1000m/z, resolution 70,000, AGC target 1e6 and maximum injection time of 80ms. All numerical and statistical data analysis were performed by Graphpad Prism 7. Confocal microscopy data analysis was performed by Zeiss Zen2012 (Blue edition).

For manuscripts utilizing custom algorithms or software that are central to the research but not yet described in published literature, software must be made available to editors and reviewers. We strongly encourage code deposition in a community repository (e.g. GitHub). See the Nature Research guidelines for submitting code & software for further information.

Data

Policy information about availability of data

All manuscripts must include a data availability statement. This statement should provide the following information, where applicable:

- Accession codes, unique identifiers, or web links for publicly available datasets
- A list of figures that have associated raw data
- A description of any restrictions on data availability

RNAseq results are available in Table S1. Metabolomic results are available in Table S2-3. We have uploaded the RNAseq dataset into the Gene Expression Omnibus (GEO) with accession # GSE161881.

Field-specific reporting

Please select the one below that is the best fit for your research. If you are not sure, read the appropriate sections before making your selection.

☒ Life sciences ☐ Behavioural & social sciences ☐ Ecological, evolutionary & environmental sciences

For a reference copy of the document with all sections, see nature.com/documents/nr-reporting-summary-flat.pdf

Life sciences study design

All studies must disclose on these points even when the disclosure is negative.

Sample size	Power calculations were performed for pairwise comparisons of independent groups of quantitative data. Using group size of at least n=3 guarantees the power for a hypothesis test at the 5% significance. 6 biological replicates were used for the metabolomics analysis.
Data exclusions	No data were excluded from the analysis.
Replication	All experiments were biologically repeated multiple times to verify the reproducibility of the results. Those results were consistent between different replicates.
Randomization	N/A. All the experiments were done in vitro.
Blinding	N/A. All the experiments were done in vitro.

Reporting for specific materials, systems and methods

We require information from authors about some types of materials, experimental systems and methods used in many studies. Here, indicate whether each material, system or method listed is relevant to your study. If you are not sure if a list item applies to your research, read the appropriate section before selecting a response.

Materials & experimental systems

n/a	Involved in the study	
☐	☒	Antibodies
☐	☒	Eukaryotic cell lines
☒	☐	Palaeontology and archaeology
☒	☐	Animals and other organisms
☒	☐	Human research participants
☒	☐	Clinical data
☒	☐	Dual use research of concern

Methods

n/a	Involved in the study	
☒	☐	ChIP-seq
☐	☒	Flow cytometry
☒	☐	MRI-based neuroimaging

Antibodies

Antibodies used	SHMT1 (#12612S), SHMT2 (#12762S) and GAPDH (#5174S) polyclonal antibodies were purchase from Cell Signaling Technology. Anti-SARS-CoV-2 nucleocapsid mouse monoclonal antibody SA46 was kindly provided by Dr. Ying Fang (University of Illinois at Urbana-Champaign).Anti-ACE2 antibody (AF933) was bought from R&D Systems, and the FITC-conjugated anti-goat secondary antibody (A21467)was bought from Invitrogen.
Validation	Antibodies against SHMT1 and SHMT2 were used in 1:1000 in WB. The second antibody was used anti-Rabbit HRP (#7074V, CST) in 1:5000. Antibody against GAPDH was used in 1:1000 in WB. The second antibody for GAPDH was anti-Mouse HPR (#7076V, CST) in 1:5000. We confirmed by western blot that a band at the expected molecular weight decreased in signal intensity upon expression of the guide RNA targeting the gene encoding the protein of interest. were well validated for western blot by Cell Signaling Technology. Anti-GAPDH antibody is widely used and was well validated for western blot by Cell Signaling Technology. Anti-SARS-CoV-2 nucleocapsid antibody SA-46 was validated by comparing the immunofluorecense signal of the SARS-CoV-2 infected cells to the mock

	infected cell. Anti-ACE2 antibody (R&D Systems, AF933) was used in 0.25 μg/10^6 cells. FITC-conjugated anti-goat secondary antibody (Invitrogen, A21467)was used in 1:1000.

Eukaryotic cell lines

Policy information about cell lines

Cell line source(s)	Vero E6 TMPRSS2+ cellwas a gift from Drs. Matteo Gentili and Nir Hacohen. It was constructed by lentiviral transduction of Vero E6 (ATCC) using the construct pTRIP-SFFV-Hygro-2A-TMPRSS2 and selection at 500μg/ml hygromycin. A549 ACE2+ cells were a gift from Colin O'Leary and Stephen Elledge.
Authentication	Parental Vero E6(CRL-1586) and A549(CCL-185) cells were validated and acquired from ATCC.
Mycoplasma contamination	Cells were certified as mycoplasma-free using the MycoAlert kit (Lonza).
Commonly misidentified lines (See ICLAC register)	No commonly misidentified lines were used in the study.

Flow Cytometry

Plots

Confirm that:

- [x] The axis labels state the marker and fluorochrome used (e.g. CD4-FITC).
- [x] The axis scales are clearly visible. Include numbers along axes only for bottom left plot of group (a 'group' is an analysis of identical markers).
- [x] All plots are contour plots with outliers or pseudocolor plots.
- [x] A numerical value for number of cells or percentage (with statistics) is provided.

Methodology

Sample preparation	See "Np sgRNA Flow-FISH" section in materials and methods
Instrument	BD FACSCalibur; BD FACSAria™ III sorter
Software	BD Cellquest Pro was used for data collection. Flowjo X was used for data analysis.
Cell population abundance	For all studies, 10000 live cells were gated. CHistogram was used for the validation studies.
Gating strategy	Gating strategy was decided use of mock infected cells. FSC/SSC gating and flow-FISH gating data were shown in Supplementary Fig. 9.

- [x] Tick this box to confirm that a figure exemplifying the gating strategy is provided in the Supplementary Information.
